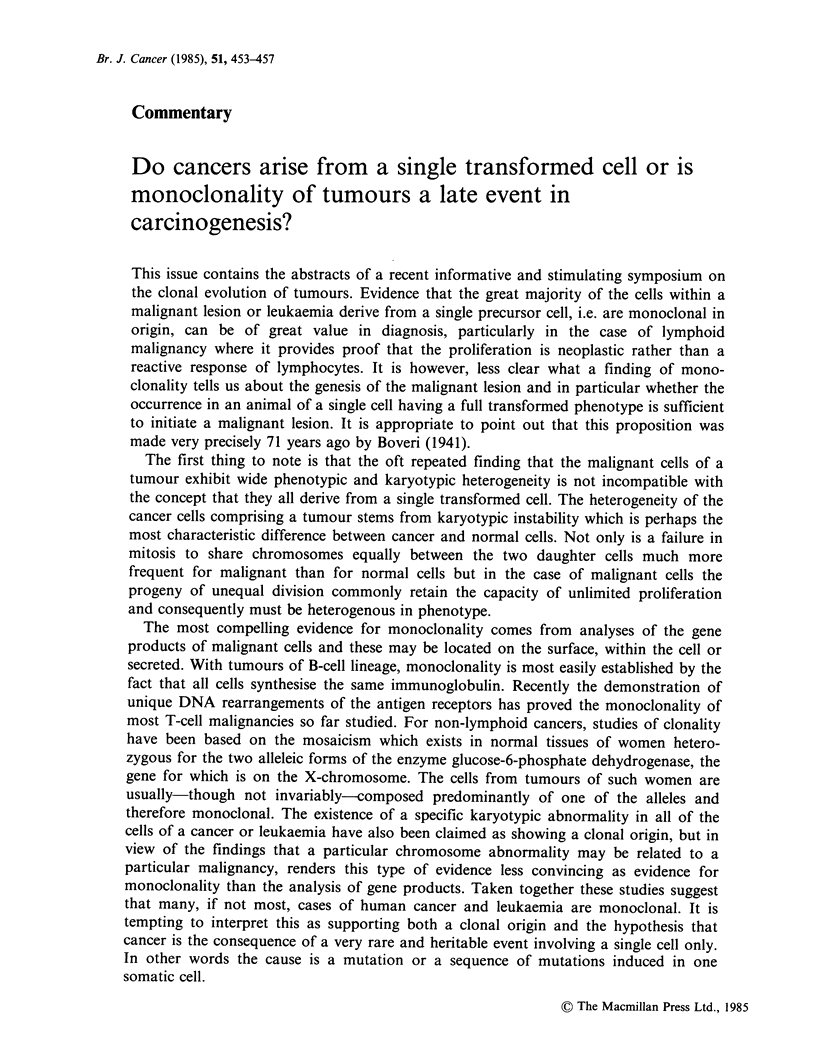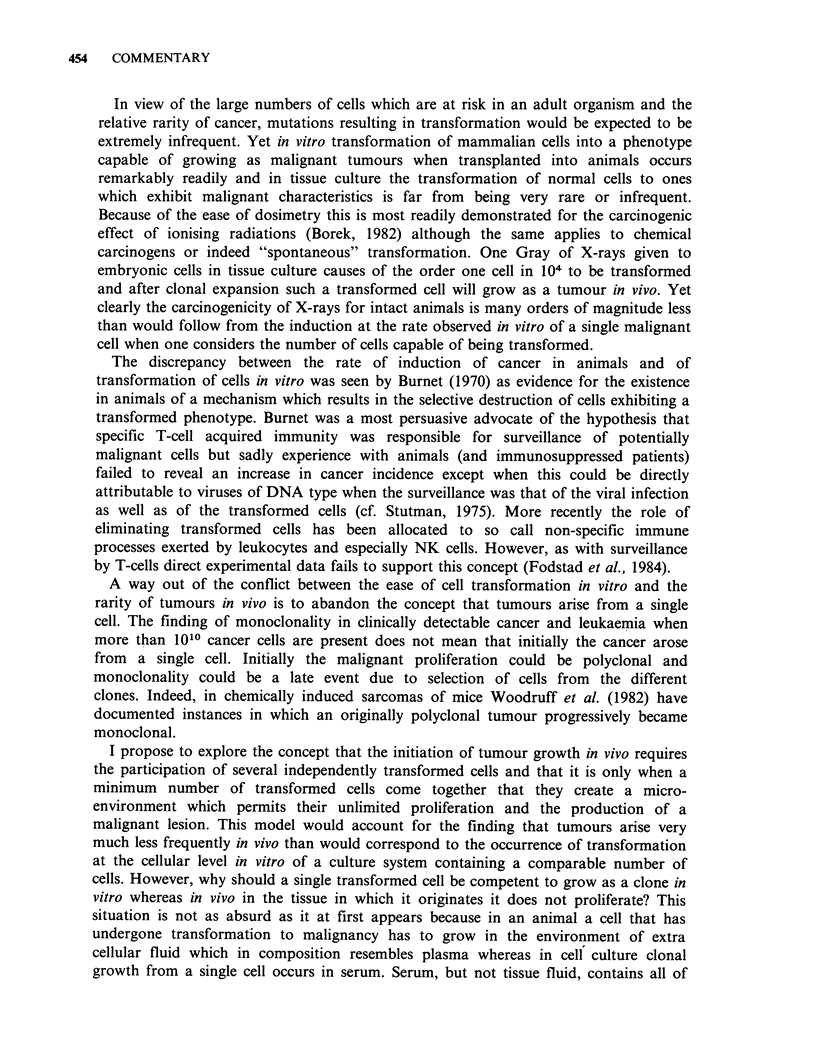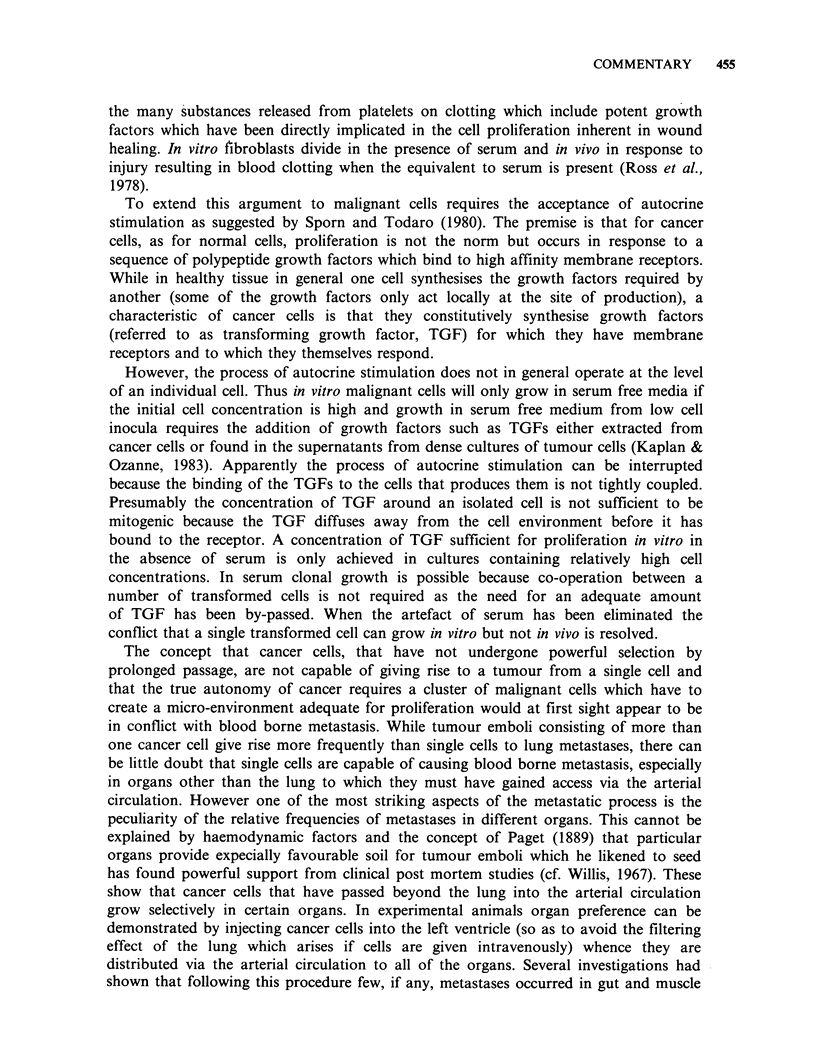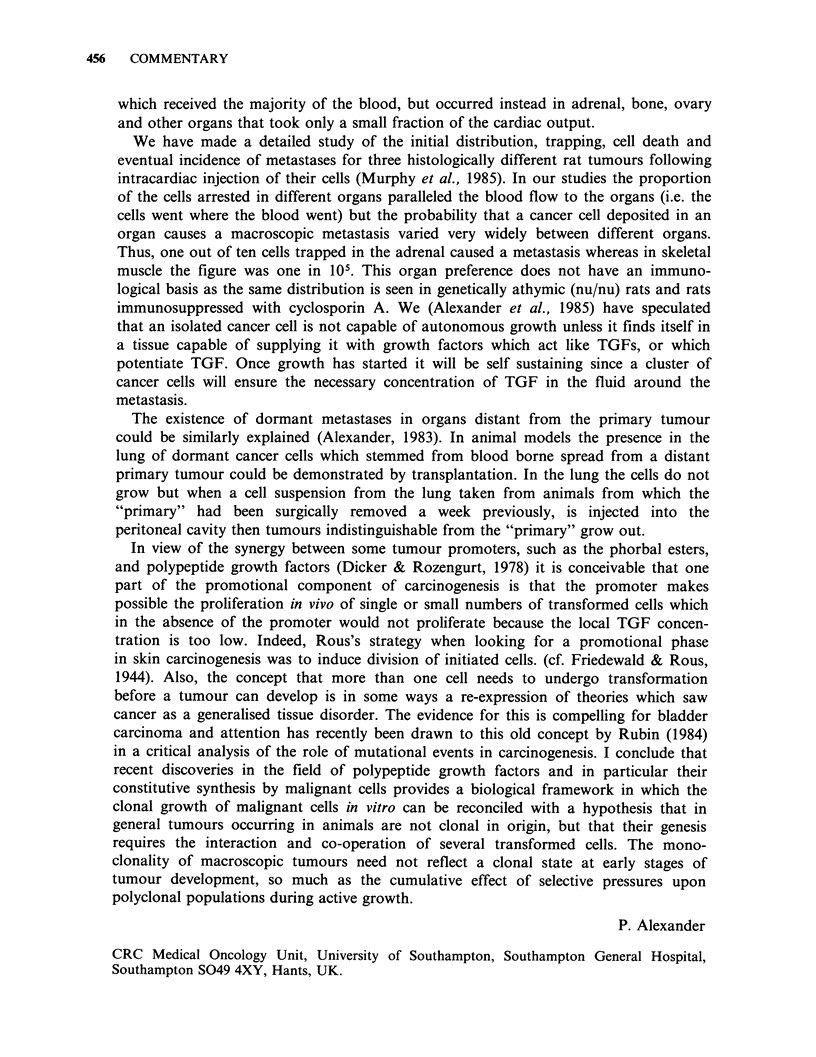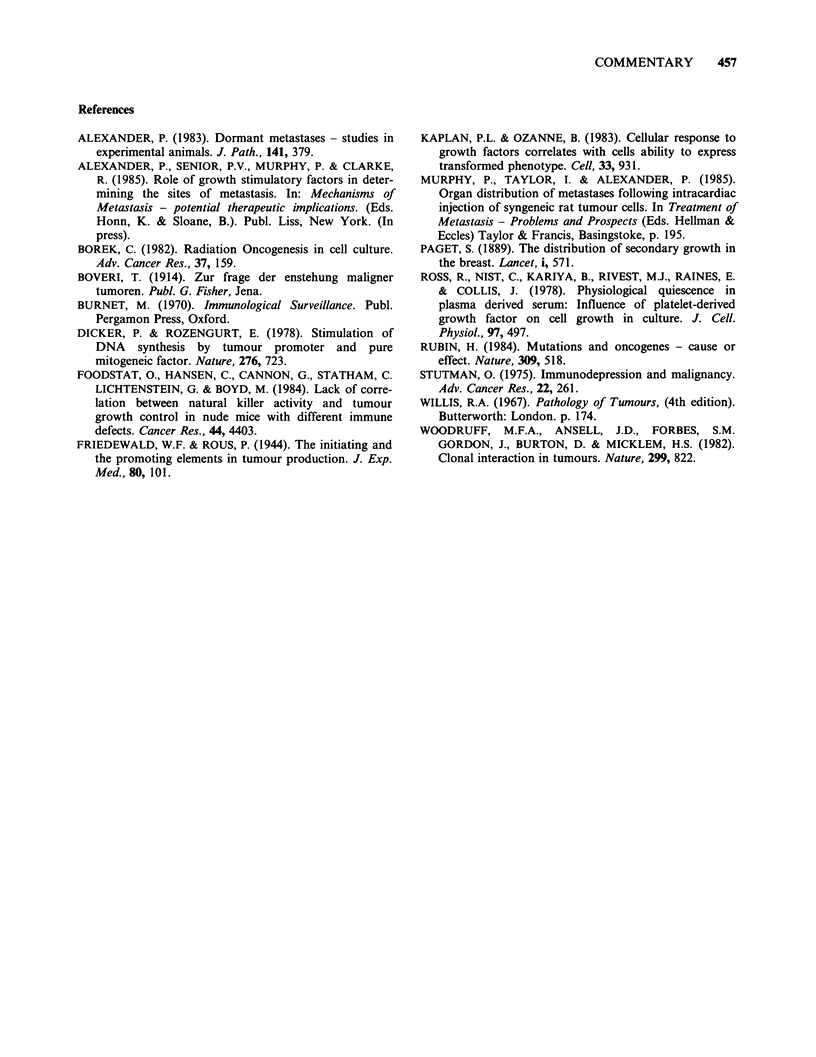# Do cancers arise from a single transformed cell or is monoclonality of tumours a late event in carcinogenesis?

**DOI:** 10.1038/bjc.1985.65

**Published:** 1985-04

**Authors:** P. Alexander


*Br. J. Cancer* (1985), **51**, 453–457

### Commentary

### Do cancers arise from a single transformed cell or is monoclonality of tumours a late event in carcinogenesis?

This issue contains the abstracts of a recent informative and stimulating symposium on the clonal evolution of tumours. Evidence that the great majority of the cells within a malignant lesion or leukaemia derive from a single precursor cell, i.e. are monoclonal in origin, can be of great value in diagnosis, particularly in the case of lymphoid malignancy where it provides proof that the proliferation is neoplastic rather than a reactive response of lymphocytes. It is however, less clear what a finding of monoclonality tells us about the genesis of the malignant lesion and in particular whether the occurrence in an animal of a single cell having a full transformed phenotype is sufficient to initiate a malignant lesion. It is appropriate to point out that this proposition was made very precisely 71 years ago by Boveri (1941).

The first thing to note is that the oft repeated finding that the malignant cells of a tumour exhibit wide phenotypic and karyotypic heterogeneity is not incompatible with the concept that they all derive from a single transformed cell. The heterogeneity of the cancer cells comprising a tumour stems from karyotypic instability which is perhaps the most characteristic difference between cancer and normal cells. Not only is a failure in mitosis to share chromosomes equally between the two daughter cells much more frequent for malignant than for normal cells but in the case of malignant cells the progeny of unequal division commonly retain the capacity of unlimited proliferation and consequently must be heterogenous in phenotype.

The most compelling evidence for monoclonality comes from analyses of the gene products of malignant cells and these may be located on the surface, within the cell or secreted. With tumours of B-cell lineage, monoclonality is most easily established by the fact that all cells synthesise the same immunoglobulin. Recently the demonstration of unique DNA rearrangements of the antigen receptors has proved the monoclonality of most T-cell malignancies so far studied. For non-lymphoid cancers, studies of clonality have been based on the mosaicism which exists in normal tissues of women heterozygous for the two alleleic forms of the enzyme glucose-6-phosphate dehydrogenase, the gene for which is on the X-chromosome. The cells from tumours of such women are usually—though not invariably—composed predominantly of one of the alleles and therefore monoclonal. The existence of a specific karyotypic abnormality in all of the cells of a cancer or leukaemia have also been claimed as showing a clonal origin, but in view of the findings that a particular chromosome abnormality may be related to a particular malignancy, renders this type of evidence less convincing as evidence for monoclonality than the analysis of gene products. Taken together these studies suggest that many, if not most, cases of human cancer and leukaemia are monoclonal. It is tempting to interpret this as supporting both a clonal origin and the hypothesis that cancer is the consequence of a very rare and heritable event involving a single cell only. In other words the cause is a mutation or a sequence of mutations induced in one somatic cell.

© The Macmillan Press Ltd., 1985

In view of the large numbers of cells which are at risk in an adult organism and the relative rarity of cancer, mutations resulting in transformation would be expected to be extremely infrequent. Yet *in vitro* transformation of mammalian cells into a phenotype capable of growing as malignant tumours when transplanted into animals occurs remarkably readily and in tissue culture the transformation of normal cells to ones which exhibit malignant characteristics is far from being very rare or infrequent. Because of the ease of dosimetry this is most readily demonstrated for the carcinogenic effect of ionising radiations (Borek, 1982) although the same applies to chemical carcinogens or indeed "spontaneous" transformation. One Gray of X-rays given to embryonic cells in tissue culture causes of the order one cell in $10^4$ to be transformed and after clonal expansion such a transformed cell will grow as a tumour *in vivo*. Yet clearly the carcinogenicity of X-rays for intact animals is many orders of magnitude less than would follow from the induction at the rate observed *in vitro* of a single malignant cell when one considers the number of cells capable of being transformed.

The discrepancy between the rate of induction of cancer in animals and of transformation of cells *in vitro* was seen by Burnet (1970) as evidence for the existence in animals of a mechanism which results in the selective destruction of cells exhibiting a transformed phenotype. Burnet was a most persuasive advocate of the hypothesis that specific T-cell acquired immunity was responsible for surveillance of potentially malignant cells but sadly experience with animals (and immunosuppressed patients) failed to reveal an increase in cancer incidence except when this could be directly attributable to viruses of DNA type when the surveillance was that of the viral infection as well as of the transformed cells (cf. Stutman, 1975). More recently the role of eliminating transformed cells has been allocated to so call non-specific immune processes exerted by leukocytes and especially NK cells. However, as with surveillance by T-cells direct experimental data fails to support this concept (Fodstad *et al.*, 1984).

A way out of the conflict between the ease of cell transformation *in vitro* and the rarity of tumours *in vivo* is to abandon the concept that tumours arise from a single cell. The finding of monoclonality in clinically detectable cancer and leukaemia when more than $10^{10}$ cancer cells are present does not mean that initially the cancer arose from a single cell. Initially the malignant proliferation could be polyclonal and monoclonality could be a late event due to selection of cells from the different clones. Indeed, in chemically induced sarcomas of mice Woodruff *et al.* (1982) have documented instances in which an originally polyclonal tumour progressively became monoclonal.

I propose to explore the concept that the initiation of tumour growth *in vivo* requires the participation of several independently transformed cells and that it is only when a minimum number of transformed cells come together that they create a micro-environment which permits their unlimited proliferation and the production of a malignant lesion. This model would account for the finding that tumours arise very much less frequently *in vivo* than would correspond to the occurrence of transformation at the cellular level *in vitro* of a culture system containing a comparable number of cells. However, why should a single transformed cell be competent to grow as a clone *in vitro* whereas *in vivo* in the tissue in which it originates it does not proliferate? This situation is not as absurd as it at first appears because in an animal a cell that has undergone transformation to malignancy has to grow in the environment of extra cellular fluid which in composition resembles plasma whereas in cell culture clonal growth from a single cell occurs in serum. Serum, but not tissue fluid, contains all of the many substances released from platelets on clotting which include potent growth factors which have been directly implicated in the cell proliferation inherent in wound healing. *In vitro* fibroblasts divide in the presence of serum and *in vivo* in response to injury resulting in blood clotting when the equivalent to serum is present (Ross *et al.*, 1978).

To extend this argument to malignant cells requires the acceptance of autocrine stimulation as suggested by Sporn and Todaro (1980). The premise is that for cancer cells, as for normal cells, proliferation is not the norm but occurs in response to a sequence of polypeptide growth factors which bind to high affinity membrane receptors. While in healthy tissue in general one cell synthesises the growth factors required by another (some of the growth factors only act locally at the site of production), a characteristic of cancer cells is that they constitutively synthesise growth factors (referred to as transforming growth factor, TGF) for which they have membrane receptors and to which they themselves respond.

However, the process of autocrine stimulation does not in general operate at the level of an individual cell. Thus *in vitro* malignant cells will only grow in serum free media if the initial cell concentration is high and growth in serum free medium from low cell inocula requires the addition of growth factors such as TGFs either extracted from cancer cells or found in the supernatants from dense cultures of tumour cells (Kaplan & Ozanne, 1983). Apparently the process of autocrine stimulation can be interrupted because the binding of the TGFs to the cells that produces them is not tightly coupled. Presumably the concentration of TGF around an isolated cell is not sufficient to be mitogenic because the TGF diffuses away from the cell environment before it has bound to the receptor. A concentration of TGF sufficient for proliferation *in vitro* in the absence of serum is only achieved in cultures containing relatively high cell concentrations. In serum clonal growth is possible because co-operation between a number of transformed cells is not required as the need for an adequate amount of TGF has been by-passed. When the artefact of serum has been eliminated the conflict that a single transformed cell can grow *in vitro* but not *in vivo* is resolved.

The concept that cancer cells, that have not undergone powerful selection by prolonged passage, are not capable of giving rise to a tumour from a single cell and that the true autonomy of cancer requires a cluster of malignant cells which have to create a micro-environment adequate for proliferation would at first sight appear to be in conflict with blood borne metastasis. While tumour emboli consisting of more than one cancer cell give rise more frequently than single cells to lung metastases, there can be little doubt that single cells are capable of causing blood borne metastasis, especially in organs other than the lung to which they must have gained access via the arterial circulation. However one of the most striking aspects of the metastatic process is the peculiarity of the relative frequencies of metastases in different organs. This cannot be explained by haemodynamic factors and the concept of Paget (1889) that particular organs provide expecially favourable soil for tumour emboli which he likened to seed has found powerful support from clinical post mortem studies (cf. Willis, 1967). These show that cancer cells that have passed beyond the lung into the arterial circulation grow selectively in certain organs. In experimental animals organ preference can be demonstrated by injecting cancer cells into the left ventricle (so as to avoid the filtering effect of the lung which arises if cells are given intravenously) whence they are distributed via the arterial circulation to all of the organs. Several investigations had shown that following this procedure few, if any, metastases occurred in gut and muscle which received the majority of the blood, but occurred instead in adrenal, bone, ovary and other organs that took only a small fraction of the cardiac output.

We have made a detailed study of the initial distribution, trapping, cell death and eventual incidence of metastases for three histologically different rat tumours following intracardiac injection of their cells (Murphy *et al.,* 1985). In our studies the proportion of the cells arrested in different organs paralleled the blood flow to the organs (i.e. the cells went where the blood went) but the probability that a cancer cell deposited in an organ causes a macroscopic metastasis varied very widely between different organs. Thus, one out of ten cells trapped in the adrenal caused a metastasis whereas in skeletal muscle the figure was one in $10^5$. This organ preference does not have an immunological basis as the same distribution is seen in genetically athymic (nu/nu) rats and rats immunosuppressed with cyclosporin A. We (Alexander *et al.,* 1985) have speculated that an isolated cancer cell is not capable of autonomous growth unless it finds itself in a tissue capable of supplying it with growth factors which act like TGFs, or which potentiate TGF. Once growth has started it will be self sustaining since a cluster of cancer cells will ensure the necessary concentration of TGF in the fluid around the metastasis.

The existence of dormant metastases in organs distant from the primary tumour could be similarly explained (Alexander, 1983). In animal models the presence in the lung of dormant cancer cells which stemmed from blood borne spread from a distant primary tumour could be demonstrated by transplantation. In the lung the cells do not grow but when a cell suspension from the lung taken from animals from which the "primary" had been surgically removed a week previously, is injected into the peritoneal cavity then tumours indistinguishable from the "primary" grow out.

In view of the synergy between some tumour promoters, such as the phorbal esters, and polypeptide growth factors (Dicker & Rozengurt, 1978) it is conceivable that one part of the promotional component of carcinogenesis is that the promoter makes possible the proliferation *in vivo* of single or small numbers of transformed cells which in the absence of the promoter would not proliferate because the local TGF concentration is too low. Indeed, Rous's strategy when looking for a promotional phase in skin carcinogenesis was to induce division of initiated cells. (cf. Friedewald & Rous, 1944). Also, the concept that more than one cell needs to undergo transformation before a tumour can develop is in some ways a re-expression of theories which saw cancer as a generalised tissue disorder. The evidence for this is compelling for bladder carcinoma and attention has recently been drawn to this old concept by Rubin (1984) in a critical analysis of the role of mutational events in carcinogenesis. I conclude that recent discoveries in the field of polypeptide growth factors and in particular their constitutive synthesis by malignant cells provides a biological framework in which the clonal growth of malignant cells *in vitro* can be reconciled with a hypothesis that in general tumours occurring in animals are not clonal in origin, but that their genesis requires the interaction and co-operation of several transformed cells. The monoclonality of macroscopic tumours need not reflect a clonal state at early stages of tumour development, so much as the cumulative effect of selective pressures upon polyclonal populations during active growth.

P. Alexander

CRC Medical Oncology Unit, University of Southampton, Southampton General Hospital, Southampton SO49 4XY, Hants, UK.

### References

ALEXANDER, P. (1983). Dormant metastases – studies in experimental animals. *J. Path.*, **141,** 379.

ALEXANDER, P., SENIOR, P.V., MURPHY, P. & CLARKE, R. (1985). Role of growth stimulatory factors in determining the sites of metastasis. In: *Mechanisms of Metastasis – potential therapeutic implications*. (Eds. Honn, K. & Sloane, B.). Publ. Liss, New York. (In press).

BOREK, C. (1982). Radiation Oncogenesis in cell culture. *Adv. Cancer Res.*, **37,** 159.

BOVERI, T. (1914). Zur frage der enstehung maligner tumoren. *Publ. G. Fisher,* Jena.

BURNET, M. (1970). *Immunological Surveillance*. Publ. Pergamon Press, Oxford.

DICKER, P. & ROZENGURT, E. (1978). Stimulation of DNA synthesis by tumour promoter and pure mitogeneic factor. *Nature,* **276,** 723.

FOODSTAT, O., HANSEN, C., CANNON, G., STATHAM, C. LICHTENSTEIN, G. & BOYD, M. (1984). Lack of correlation between natural killer activity and tumour growth control in nude mice with different immune defects. *Cancer Res.*, **44,** 4403.

FRIEDEWALD, W.F. & ROUS, P. (1944). The initiating and the promoting elements in tumour production. *J. Exp. Med.*, **80,** 101.

KAPLAN, P.L. & OZANNE, B. (1983). Cellular response to growth factors correlates with cells ability to express transformed phenotype. *Cell,* **33,** 931.

MURPHY, P., TAYLOR, I. & ALEXANDER, P. (1985). Organ distribution of metastases following intracardiac injection of syngeneic rat tumour cells. In *Treatment of Metastasis – Problems and Prospects* (Eds. Hellman & Eccles) Taylor & Francis, Basingstoke, p. 195.

PAGET, S. (1889). The distribution of secondary growth in the breast. *Lancet,* **i,** 571.

ROSS, R., NIST, C., KARIYA, B., RIVEST, M.J., RAINES, E. & COLLIS, J. (1978). Physiological quiescence in plasma derived serum: Influence of platelet-derived growth factor on cell growth in culture. *J. Cell. Physiol.*, **97,** 497.

RUBIN, H. (1984). Mutations and oncogenes – cause or effect. *Nature,* **309,** 518.

STUTMAN, O. (1975). Immunodepression and malignancy. *Adv. Cancer Res.*, **22,** 261.

WILLIS, R.A. (1967). *Pathology of Tumours,* (4th edition). Butterworth: London. p. 174.

WOODRUFF, M.F.A., ANSELL, J.D., FORBES, S.M. GORDON, J., BURTON, D. & MICKLEM, H.S. (1982). Clonal interaction in tumours. *Nature,* **299,** 822.